# Pharmacist-led medication reconciliation at patient discharge: a tool to reduce healthcare utilization? an observational study in patients 65 years or older

**DOI:** 10.1186/s12877-022-03192-3

**Published:** 2022-07-13

**Authors:** Emma Bajeux, Lilian Alix, Lucie Cornée, Camille Barbazan, Marion Mercerolle, Jennifer Howlett, Vincent Cruveilhier, Charlotte Liné-Iehl, Bérangère Cador, Patrick Jego, Vincent Gicquel, François-Xavier Schweyer, Vanessa Marie, Stéphanie Hamonic, Jean-Michel Josselin, Dominique Somme, Benoit Hue

**Affiliations:** 1 Department of Epidemiology and Public Health, Univ Rennes, Rennes University Hospital, F-35000 Rennes, France; 2 Department of Internal Medicine and Clinical Immunology, Univ Rennes, Rennes University Hospital, F-35000 Rennes, France; 3 Department of Geriatrics, St-Laurent Polyclinic, Hospitalité St-Thomas de Villeneuve, F-35000 Rennes, France; 4 Department of Pharmacy, Rennes University Hospital, F-35000 Rennes, France; 5 Department of Pharmacy, Fougères Hospital, F-35300 Fougères, France; 6 Department of Pharmacy, Montfort/Meu Hospital, F-35160 Montfort/Meu, France; 7 Department of Human and Social Sciences, Univ Rennes, EHESP, EA7348 MOS, F-35000 Rennes, France; 8 France Asso Santé, F-35000 Rennes, France; 9 Univ Rennes, CNRS, CREM-UMR 6211, F-35000 Rennes, France; 10 Department of Geriatrics, Department of Geriatrics, Univ Rennes, EHESP, CNRS, Inserm, Arènes - UMR 6051, RSMS - U 1309 , F-35000 Rennes, France

**Keywords:** Medication Reconciliation, Adverse Drug Event, Older people, Discharge, Experience

## Abstract

**Background:**

Older patients often experience adverse drug events (ADEs) after discharge that may lead to unplanned readmission. Medication Reconciliation (MR) reduces medication errors that lead to ADEs, but results on healthcare utilization are still controversial. This study aimed to assess the effect of MR at discharge (MRd) provided to patients aged over 65 on their unplanned rehospitalization within 30 days and on both patients’ experience of discharge and their knowledge of their medication.

**Methods:**

An observational multicenter prospective study was conducted in 5 hospitals in Brittany, France.

**Results:**

Patients who received both MR on admission (MRa) and MRd did not have significantly fewer deaths, unplanned rehospitalizations and/or emergency visits related to ADEs (OR = 1.6 [0.7 to 3.6]) or whatever the cause (*p* = 0.960) 30 days after discharge than patients receiving MRa alone. However, patients receiving both MRa and MRd were more likely to feel that their discharge from the hospital was well organized (*p* = 0.003) and reported more frequently that their community pharmacist received information about their hospital stay (*p* = 0.036).

**Conclusions:**

This study found no effect of MRd on healthcare utilization 30 days after discharge in patients over 65, but the process improved patients’ experiences of care continuity. Further studies are needed to better understand this positive impact on their drug care pathway in order to improve patients’ ownership of their drugs, which is still insufficient. Improving both the interview step between pharmacist and patient before discharge and the transmission of information from the hospital to primary care professionals is needed to enhance MR effectiveness.

**Trial registration:**

NCT04018781 July 15, 2019.

**Supplementary Information:**

The online version contains supplementary material available at 10.1186/s12877-022-03192-3.

## Background

Care transition after hospitalization, when the patient is back home, is a high-risk period for adverse events, many of which are due to drug regimens [1–3]. Such adverse drug events (ADEs) can cause serious harm to patient health and are a considerable economic burden on the health care system [4, 5]. Some of them lead to emergency visits or rehospitalization as an estimated 4.5% to 24% of hospital readmissions are drug-related [6–8].

Older patients are particularly predisposed to ADEs because of altered pharmacokinetics and pharmacodynamics, multimorbidity, higher frailty and polypharmacy – usually defined as five or more medications daily – with a higher risk of drug interactions [9, 10]. Moreover, hospitalization amplifies this risk as it often induces a discontinuity considering the medical pathway care for older people because of many drug changes [10, 11].

ADEs could be caused by adverse drug reaction or to medication errors that can be prevented. [1, 2, 6]. Medication errors can notably result from an inappropriate dosage for the older patient's frailty or low weight, the inappropriate interruption of usual medication on patient admission, erroneous change of doses or galenic formulations or administration modalities, duplication of active substance, undue continuation or addition of a new medication or failure to resume the patient's usual treatments on discharge, nonadherence or lack of monitoring [12, 13].

Consequently, many hospital pharmacist interventions have been implemented to reduce the occurrence of medication errors and ADEs after discharge. The Medication Reconciliation (MR) process has gradually spread worldwide over the last 20 years and has been acknowledged by many national patient safety organizations as a way to improve medication safety at the transition points in the care pathway. The French National Authority for Health (NHA) published its first recommendations for MR in health facilities in 2016 and updated them in 2018 [12]. MR was defined as a formalized process that takes into account all the drugs taken and to be taken by the patient when drafting a new prescription. It involves the patient or the person responsible for the patient's medication (partner or relatives, health professionals that know the patient) and is based on information sharing and multi-professional coordination. The objective is to prevent and correct medication errors, by promoting the transmission of complete and accurate information on the patient's medication among healthcare professionals at each transition point in the care pathway, i.e. admission and discharge [12]. Two stages can be distinguished for MR at hospital, namely MR at admission (MRa), which draws up a detailed inventory of the patient's usual medicines, and MR at discharge (MRd). MRd consists of a new exhaustive pharmaceutical analysis, a collaborative interview between the hospital physician and the hospital pharmacist as well as a patient interview by the hospital pharmacist in a therapeutic education approach to empower the patient and to optimize the chances of maintaining the adaptation of the therapeutic regimen carried out during the hospital stay.

Most previous studies have shown an impact of the MR process on medication errors [14, 15], but its impact on the reduction of readmission, for whatever the reasons or for ADEs, and of emergency visits remains controversial [15–24]. Studies specially focused on people aged 65 or more found similar results [15, 25–28]. The results of these interventions appear to be highly variable and dependent on the detailed nature of the intervention (MRa and/or MRd, sometimes not well documented, with varying definitions from one country to another), on the health system and on the primary endpoint. Few studies have specifically assessed the effect of the MRd process, mostly only on medication errors, and there are no data on older people [13, 23, 29–32].

The aim of this study was to assess the impact of MRd to patients aged 65 or older on healthcare utilization for ADEs within 30 days following the discharge.

## Methods

### Study design

The CONPARMED study^1^ was based on a call for projects funded by the French Ministry of Health aimed at developing and at assessing clinical pharmacy inside healthcare facilities. An observational, prospective, multicenter study was set up in 5 French hospitals in 2019. This study aimed to compare two groups of patients in routine healthcare practice, those receiving a full MR process including MRa and MRd – “MRa and MRd group” — and patients receiving a partial MR process with MRa only – “MRa only group” – during a hospital stay, on the basis of design methodological choices relating to pragmatic studies [33–35]. Indeed, in a pragmatic study, the paradigm is that it is inevitable that there are some discrepancies between what is ideally planned and what is actually delivered. The purpose is to record all these discrepancies and use it as explaining factors of outcomes of interest.

### Setting and participants

The inclusion criteria were patients 65 years or older having received MRa in one of the 12 wards involved in the study – 7 medical wards (2 departments of internal medicine, 2 departments of geriatrics, 1 of general medicine and 2 of pulmonology) and 5 rehabilitation wards^2^—in 5 hospitals including 1 university hospital and 4 standard hospitals. All 5 hospitals were located in the same territorial hospital group. Such groups were created in 2016 throughout France in order to improve coordination between public hospital care providers. These hospital groups were centered around a support facility for a defined geographical area so as to facilitate joint and graduated patient care in intra-hospital care systems**,** formalized in a joint medical and nursing project [36]. The exclusion criteria were: patients in palliative care, those who died during hospitalization, those transferred to a second medical ward after the first ward in which the inclusion was carried out, and patients still hospitalized at the end of the follow-up study period. Due to the timetable imposed by the study's funder—the French Ministry of Solidarity and Health—inclusions started in June 2019 and were stopped in November 2019.

### Medication reconciliation process

**Fig. 1:**
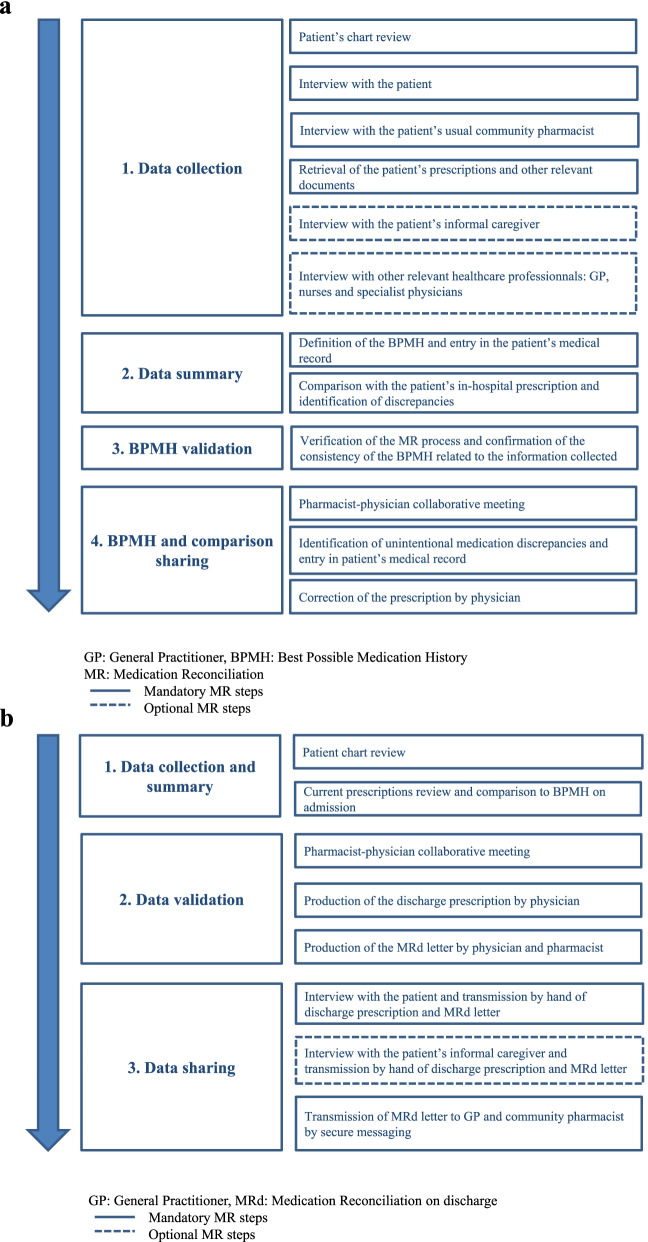
**a** Description of the steps in the Medication Reconciliation procedure on admission. **b** Description of the steps in the Medication Reconciliation procedure on discharge

In this study, the MRa and MRd processes were conducted in four steps according the recommendations of the NHA: 1) Data collection (collect the current medication list); 2) Data summary (synthesis of the Best Possible Medication History and comparison with the patient’s current prescription); 3) Best Possible Medication History validation (verification of the process by a pharmacist or physician), and 4) Information sharing with the general practitioner and community pharmacist by secure e-mail and with the patient him/herself and if applicable his/her caregiver during an interview [12]. The detailed stages of MRa and MRd are described in Figs. 1a and 1b, respectively. All steps were carried out by hospital pharmacists. Details about duration of each steps are available in a previous study [37].

As part of the patient’s usual care, the MR process was associated with the pharmaceutical analysis of the prescriptions during the hospital stay where physicians and pharmacists discuss in order to choose the most accurate and relevant medication.

### Data collection

At inclusion, at the beginning of hospitalization, patients completed self-reported questionnaires on their demographic and socioeconomic characteristics as well as their home care services, if any. The pharmacists detailed the MRa procedure as it had actually been carried out, the patient's health status as well as medications taken at admission. Cognitive impairment was defined by a previous diagnosis of dementia and/or cognitive impairment noted by the hospital pharmacist during the MRa. At the time of discharge, it was planned that all patients should undergone an MRd, but it was sometimes impossible. The MRd process was detailed in the “MRa and MRd group” while in the “MRa only group” the reasons why MRd was not conducted were recorded. The pharmacists also identified the person responsible for the patient's medication (patient himself or herself, partner or relatives, nurses operating in private practice or in nursing home). This person was called by phone 7 and 30 days after discharge by medical and pharmacist students, previously trained to assess his or her experience of the discharge and their knowledge and empowerment of the medication. A questionnaire completion guide was developed to standardize the interviewers' responses. The interviewer was blinded to the patient's arm. In addition, the call at day 30 identified whether any unplanned rehospitalization, emergency or GP visit had occurred.

### Outcomes

The primary endpoint was the proportion of patients experiencing death, unplanned rehospitalization or visit to an emergency department for ADEs within 30 days following the discharge, whichever event came first. An ADE was defined as an event with a consequence for the patient's state of health that may result from appropriate, inappropriate or absent use of a drug. Under this definition, the term ADE includes harm caused by the drug during its appropriate use (adverse drug reactions as side effects) that is not preventable and harm from the misuse of the drug (including overuse or underuse) that results from medication errors and is considered as preventable [38–40].

When a patient included in a medical ward was then admitted to a rehabilitation ward, the primary endpoint was assessed 30 days after discharge from the rehabilitation ward. When unplanned rehospitalization or an emergency visit was reported by the person responsible for the patient's medication, investigators collected hospitalization and emergency records, laboratory or imaging results as well as the discharge prescription. When information on death, unplanned rehospitalization or emergency visit were missing at day 30 (patient lost to follow-up, refusal to answer…), the investigators searched for a possible death, unplanned rehospitalization or emergency visit in the university hospital and inclusion ward patient records.

Then, an expert committee adjudicated whether rehospitalization or emergency visit could be linked to an ADE. Four experts made up the adjudication committee: one geriatrician (LC), one internal medicine physician (PJ), one pulmonologist (CB) and one hospital pharmacist (AR) with at least 3 years of experience of care (range 3–30 years) and specific experience in geriatrics i.e. for age-related changes, dosages adjustment and STOPP START criteria [41]. Experts blinded to the patient’s group determined retrospectively the likelihood that the event was drug-related. They were asked to rate each event as: (A-probable) "I consider that hospitalization is most likely related to an ADE", (B-unlikely) "I consider that hospitalization is not linked to an ADE" and (C-doubtful) "I consider that hospitalization cannot be classified as either A or B”. First, each expert had to independently classify the event. When all four experts were unanimous, their answer was recorded. Answer (A) was also recorded when three of out of the four experts classified the event in (A) and the fourth in (B). For all other cases, discrepancies were resolved by deliberation and consensus between the four experts.

Secondary endpoints were unplanned rehospitalization or any visit to an emergency department whatever the reasons and the patient’s experience of the discharge and their knowledge and appropriation of their medication, 7 and 30 days after discharge.

### Statistical analysis

Rehospitalization rates of patients aged 65 years or more are estimated at around 15% in France [42]. To our knowledge, there is no available data on rehospitalization rates on patients who received only MRa and not MRd during their hospital stay. We hypothesized an absolute reduction of this rate by 3% when MRa is done, so the sample size was based on the assumption of 12% of patients presented the primary endpoint in the “MRa only” group at day 30. We assumed a reduction in re-hospitalization for ADEs from 12 to 4% at 30 days, according to the results of a meta-analysis published in 2016 [15]. Assuming a two-sided α of 0.05, a power of 80%, and a 10% dropout rate, the required sample size was 470 patients (nQuery®).

Baseline clinical and demographic characteristics were described for the global population and compared according to the intervention group by chi-square tests for categorical variables and Student’s t test (or the Wilcoxon-Mann–Whitney test according to validity conditions) for continuous variables.

The primary endpoint was defined as the composite of all-cause death and ADE-related rehospitalization (including both probable and doubtful categories at day 30). We compared the proportion of patients who met the primary endpoint between the study groups, with a chi-square test. A logistic regression model was then used to identify factors linked to the primary outcome, including patient group as well as potential confounding factors (age, gender, living alone at home, education level, monthly household income, cognition status, person responsible for medication at home, self-reported health status, number and type of drugs per patient on BPMH, inclusion in a medical or rehabilitation ward and transfer from another hospital). Factors finally introduced in the model were selected according to a forward procedure. Results were expressed as odds ratios (ORs) with 95% confidence intervals (CIs). Sensitivity analysis was conducted excluding patients classified in the doubtful ADE by the experts of the adjudication committee from the statistical population. All reported *p-*values are based on two-tailed tests of significance. All the analyses were performed using SAS statistical software (SAS version 9.4 SAS Institute, Cary, NC, USA).

## Results

### Population characteristics

**Fig. 2:**
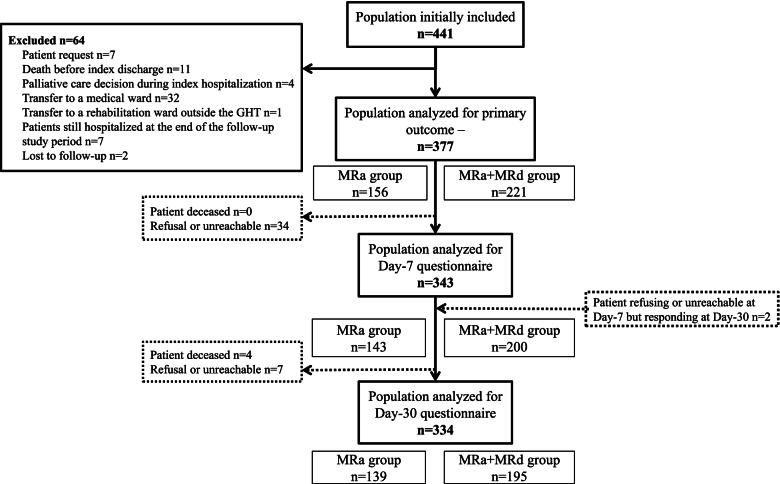
Flowchart. *MRa* Medication Reconciliation on admission, *MRd* Medication Reconciliation on discharge

Of 3,557 patients aged 65 or more, hospitalized in the study wards during the study period, 441 were initially included and 64 were excluded, leaving 377 patients in the analysis population (Fig. 2). Those 377 patients were more frequently admitted in a medical ward than in a rehabilitation ward (77.2% vs 22.8%). Among 291 patients admitted in a medical ward, 16.5% were then transferred to a rehabilitation ward. The patients came from their usual residence in 71.5% of cases and were transferred from another hospital center (in which MR was not carried out) in 28.5% of cases.

During their hospital stay, 41.4% patients received only MRa (*n *= 156) and 58.6% both MRa and MRd (*n* = 221). Demographic, socioeconomic and clinical baseline characteristics were similar in the two groups (Table 1). The drugs taken by patients on admission to the inclusion ward were more frequently cardiac therapies (76.4%), analgesics (62.3%), psychoactive drugs (46.4%), anti-thrombotic agents (44.0%) and drugs for acid suppression (35.0%) **(**Supplemental Table 1**)**. MRd was more frequently conducted for patients included in a rehabilitation ward than in a medical ward (76.7% vs 53.3%). Reasons for non-implementation of the MRd process reported by the pharmacist were: pharmacist not notified of the patient's discharge (*n* = 52), pharmacist lacked time (*n* = 44), patient discharged outside the pharmacist's working hours (*n* = 27), absence of pharmacist dedicated to MR in the hospital (*n* = 26), hospital practitioner not available to carry out the MRd with the pharmacist (*n* = 3) or unknown (*n* = 5).

**Table 1:** Patient baseline characteristics

	**Total *n* = 377**	**MRa only group *n* = 156**	**MRa and MRd group *n* = 221**	***p*-value**
**Age** Mean (SD)	81.8 (7.7)	81.7 (7.4)	81.9 (7.8)	0.732
**Gender** (Female)	214 (56.8%)	86 (55.1%)	128 (57.9%)	0.590
**Living**				
Alone at home	158 (42.8%)	62 (40.03%)	96 (44.7%)	0.400
With someone at home or in nursing homes	211 (57.2%)	92 (59.7%)	119 (55.3%)	
**Education level**				
Primary school	62 (16.8%)	21 (13.8%)	41 (19.0%)	0.583
Secondary school	256 (69.6%)	111 (73.0%)	145 (67.1%)	
High school diploma (baccalauréat)	19 (5.2%)	8 (5.3%)	11 (5.1%)	
Higher education	31 (8.4%)	12 (7.9%)	19 (8.8%)	
**Household income**				
< 500€/month	21 (6.9%)	8 (6.7%)	13 (7.0%)	0.074
[500–1,000[€/month	108 (35.4%)	51 (42.5%)	57 (30.8%)	
[1,000–2,000[€/month	139 (46.6%)	44 (36.7%)	95 (51.4%)	
> 2,000€/month	37 (12.1%)	17 (14.2%)	20 (10.8%)	
**Cognition** (Impaired)	82 (21.8%)	32 (20.6%)	50 (22.6%)	0.647
**Person responsible for medication at home** (informal or professional caregiver)	135 (36.0%)	57 (36.8%)	78 (35.5%)	0.793
**Self-reported health status** (bad or very bad)	119 (33.1%)	51 (34.7%)	68 (31.9%)	0.583
**Medication at admission** Mean (SD)	8.2 (4.0)	8.4 (4.0)	8.1 (4.1)	0.406

*SD* Standard deviation

Values are given as *N* (%) unless noted otherwise

Missing data: age (*n* = 2), living (*n* = 8), education level (*n* = 9), household income (*n* = 72), cognition (*n* = 1), person responsible for medication at home (*n* = 2), self-reported health status (*n* = 17)

### Medication reconciliation

MRa was performed retroactively in most cases, i.e., after the hospital physician had written admission orders (97.9%). The median time between admission and the completion of MRa was 1 day (interquartile range (IQR) = 1.0–3.0). Information about whether or not each step of both the MRa and MRd processes has actually been carried out and which healthcare professional has performed these steps are described in Supplemental Tables 2 and 3. The various steps of the MRa were carried out in almost all cases except the pharmacist-physician collaborative meeting, which was held in 75% of cases. For the MRd, the data collection, summary and validation steps were almost systematically conducted, while the patient (and informal caregiver interview if applicable) interview was only carried out in three-quarters of the cases. The MRd letter was sent to the GP and community pharmacist (CP) in 87.3% and 79.2% of cases, respectively.

### Primary and secondary outcomes

There was no significant difference between the two groups in terms of proportion of death, unplanned rehospitalization and/or emergency visit for ADEs, considering probable and doubtful ADEs (5.8% and 9.0% for “MRa only group” and “MRa and MRd group”, respectively; *p* = 0.239) (Table 2). Multivariate analysis did not reveal any significant association between having MRd and the primary endpoint (OR = 1.6 [0.7 – 3.6]) (Table 3). Sensitivity analysis excluding doubtful ADEs from the analysis population did not find any statistical difference either (OR = 1.4 [0.5 – 3.9]). There was no significant difference between the two groups in terms of proportion of death, unplanned rehospitalization and/or emergency visit whatever the reason (*p* = 0.960) or in the number of visits to a GP at day 30 (*p* = 0.810) (Table 2). The difference remained non-significant after stratification on rehospitalization between discharge and day 30 (*p* = 0.473).

**Table 2:** Summary of outcome at day 30 post discharge

	**Total *n* = 377**	**MRa only Group *n* = 156**	**MRa and MRd group *n* = 221**	***p*-value**
**Primary endpoint**				
Death or unplanned rehospitalization or emergency visit for ADEs (probable or doubtful)	29 (7.7%)	9 (5.8%)	20 (9.0%)	0.239
Unplanned rehospitalization or emergency visit for ADEs				
Probable	15 (4.0%)	4 (2.6%)	11 (5.0%)	
Doubtful	11 (2.9%)	3 (1.9%)	8 (3.6%)	
Unlikely	30 (8.0%)	16 (10.3%)	14 (6.3%)	
Death without any rehospitalization	3 (0.8%)	2 (1.3%)	1 (0.5%)	
**Secondary endpoints**				
Unplanned rehospitalization or emergency visit, whatever the reasons^a^	56 (14.9%)	23 (14.7%)	33 (14.9%)	0.960
GP visit				
0 visit	82 (25.5%)	33 (24.6%)	49 (26.2%)	0.810
1 visit	159 (49.5%)	64 (47.8%)	95 (50.8%)	
2 visits	55 (17.1%)	26 (19.4%)	29 (15.5%)	
≥ 3 visits	25 (7.8%)	11 (8.2%)	14 (7.5%)	

Missing data: GP visit (*n* = 56)

^a^ Including one patient who died after rehospitalization

*ADE* Adverse drug event

**Table 3:** Factors associated with death, unplanned rehospitalization or emergency visit for ADEs within 30 days post-discharge

	**Death or unplanned rehospitalization or emergency visit for ADE within 30 days post discharge**^**a**^	
	Univariate analysis OR [95%CI]	Multivariate analysis^b^ OR [95%CI]
**Drugs on BPMH (ref: no)**		
Chronic obstructive pulmonary disease drugs	2.6 [1.2- 6.0]	2.4 [1.0–5.5]
Alpha-adrenergic receptor blockers	3.3 [1.4 – 8.0]	2.7 [1.1–6.8]
Immunosuppressive agents or immunostimulants	4.9 [1.2 – 19.6]	4.1 [1.0 – 17.0]
**MRa and MRd (ref: MRa only)**	1.6 [0.7- 3.7]	1.6 [0.7–3.6]

*OR* Odds Ratio, *CI* Confidence Interval, *MRa* Medication Reconciliation on admission, *MRd* Medication Reconciliation on discharge, *ADE* Adverse Drug Event, *BPMH* Best Possible Medication History

^a^ Including A-Probable et C-Doubtful link between drugs and unplanned rehospitalization or emergency visit

^b^All variables of the bivariate analysis were introduced into the model and then selected according to a forward procedure

In the “MRa and MRd group”, only 74.9% of patients actually had the pharmaceutical interview before discharge that is part of the MRd (Supplemental Table 3) and this variable was not associated with the primary endpoint (*p* = 0.183).

Results regarding patients’ experience of discharge and their knowledge and appropriation of the medication are shown in Table 4. There was no difference between the two groups concerning the patient’s knowledge of his/her medications when leaving hospital or whether she/he had a question about the medications after discharge or not. Only 47.8% of patients who actually received MRd including the interview with the pharmacist reported talking with a health professional about their medication (vs. 33.3% of patients who did not receive MRd) and 66.5% did not remember being given a document other than a prescription setting out their medication and the changes made during their hospital stay. Patients in the “MRa and MRd group” were more likely to feel that their discharge from hospital was well organized (*p* < 0.001) and reported more frequently that their CP received information about their hospital stay (*p* = 0.003).

**Table 4:** Summary of outcome at days 7 and 30 post discharge

	**Total *n* = 343**	**MRa only Group *n* = 143**	**MRa and MRd group *n* = 200**	***p*-value**
**Phone call 7 days post-discharge**				
How was your discharge from hospital?				
Quite or very good	305 (93.3%)	128 (90.8%)	177 (95.2%)	0.117
Quite or very bad	22 (6.7%)	13 (9.2%)	9 (4.8%)	
Were you familiarized with your medications when you left the hospital?				
Yes, absolutely	224 (67.3%)	91 (65.0%)	133 (68.9%)	0.474
Yes, partially	50 (15.0%)	20 (14.3%)	30 (15.5%)	
No	59 (17.7%)	29 (20.7%)	30 (15.5%)	
During your hospitalization, did you meet with a professional to talk about your medications?				
Yes	121 (38.9%)	44 (33.3%)	77 (43.0%)	0.083
No	190 (61.1%)	88 (66.7%)	102 (57.0%)	
At the end of your hospitalization, were you given a document (other than a prescription) setting out your medication and the changes made during your hospital stay?				
Yes	70 (25.6%)	16 (14.0%)	54 (34.0%)	< 0.001***
No	203 (74.4%)	98 (86.0%)	105 (66.0%)	
After discharge, did you know who to contact if you had a question about your medication?				
Yes	273 (80.5%)	109 (77.9%)	164 (82.4%)	0.297
No	66 (19.5%)	31 (22.1%)	35 (17.6%)	
Has your GP received any information about your hospital stay?				
Yes	82 (76.6%)	42 (77.8%)	40 (75.5%)	0.778
No	25 (23.4%)	12 (22.2%)	13 (24.5%)	
Has your CP received any information about your hospital stay?				
Yes	58 (43.0%)	21 (35.0%)	37 (49.3%)	0.095
No	77 (57.0%)	39 (65.0%)	38 (50.7%)	
Did you feel there was a problem with the transmission of information between the hospital and both GP and CP?				
Yes	29 (12.4%)	17 (16.5%)	12 (9.2%)	0.090
No	205 (87.6%)	86 (83.5%)	119 (90.8%)	
Did you feel that your discharge from the hospital was well organized?				
Yes, absolutely	248 (75.4%)	92 (67.6%)	156 (80.8%)	0.003**
Yes, quite well	59 (17.9%)	28 (20.6%)	31 (16.1%)	
No	22 (6.7%)	16 (11.8%)	6 (3.1%)	
**Phone call 30 days post-discharge**				
Has your GP received any information about your hospital stay?				
Yes	210 (88.6%)	86 (85.1%)	124 (91.2%)	0.149
No	27 (11.4%)	15 (14.9%)	12 (8.8%)	
Has your CP received any information about your hospital stay?				
Yes	42 (38.9%)	13 (27.7%)	29 (47.5%)	0.036*
No	66 (61.1%)	34 (72.3%)	32 (52.5%)	
Did you feel there was a problem with the transmission of information between the hospital and both GP and CP?				
Yes	42 (19.2%)	16 (17.2%)	26 (20.6%)	0.524
No	177 (80.8%)	77 (82.8%)	100 (79.4%)	

Percentages were calculated excluding missing data or response “I don’t remember” or “I don’t know”

*CP* Community Pharmacist, *GP* General Practitioner

^***^ *p* < 0.001 ** *p* < 0.01 * *p* < 0.05

## Discussion

### Main findings

In this observational study, we found that patients who received both MRa and MRd did not have significantly fewer deaths, unplanned rehospitalizations and/or emergency visits related to ADEs, whatever the cause, or GP visits 30 days after discharge than patients receiving MRa alone. There was no difference between the two groups concerning the patient’s knowledge of his/her medications when leaving hospital. However, patients in the “MRa and MRd group” were more likely to feel that their discharge from hospital was well organized and reported more frequently that their CP received information about their hospital stay.

While there seems to be a consensus about the effects of MR on reducing medication errors, our results are in line with recent systematic reviews and meta-analyses of randomized clinical trials that did not find any significant results on readmission post-discharge [18, 21, 22], with one exception [17]. Indeed, few medication errors have the potential to lead to harm to patients and to increase healthcare utilization [43]. Furthermore, unlike other recent studies that compared the complete process of MR (MRa and MRd) to standard care i.e. without any step of the MR, all the patients included in our study underwent MRa, which could reduce the potential difference between the two groups in the primary outcome [15–24].

However, according to a previous study, our results suggested that MR may improve the patient’s experience of discharge and further studies may be needed to better understand this positive impact on the drug care pathway [44].

### Limits of the MR process in a real-life context and potential improvements

In view of the controversial results concerning the impact of the MR on healthcare utilization, it seems important to understand how the different steps are carried out, their specific effects and the ways to improve them [45]. The fact that our study was implemented in a real-life context is of interest in various ways. First, we had to check whether MRd implemented in a real-life context was conducted in accordance with NHA recommendations [12]. Among the patients who received MRd, patient interview at discharge was actually performed in only 3 out of 4 cases and MRd letter was transmitted by e-mail to GPs and CPs in very few cases. This highlights difficulties in fully implementing a long and complex MR process involving many actors and may partially explain why a potential clinical impact of MR is difficult to highlight. Furthermore, the various steps of MRd actually implemented may not be optimal and fully adapted to patients' needs. For instance, one-third of the patients did not or partially knew their medications when they left hospital, regardless of whether or not MRd was performed. Less than half of the patients who had an interview with a pharmacist reported talking with a health professional about their medication and most of them did not remember being given a document other than a prescription setting out their medication and the changes made during their hospital stay. This suggests that the day of discharge from hospital may not be the most appropriate time to perform the pharmacist interview, because patients and their caregivers receive a lot of information and documents at discharge and are probably not very receptive. Moreover, low cognition at discharge is common among older patients without dementia, and cognition often improves one month post-hospitalization [46]. Some interventions in other countries have proposed an interview a few days to a few weeks after discharge, with significant results on the rate of preventable ADEs and healthcare utilization 30 days after discharge [13, 47] and on patient satisfaction [48]. This raises the questions of who should be in charge of this interview and of the potential role of the CP who is familiar to the patient, but this implies an effective transmission of information from the hospital to the CP, which was not the case in our study.

The implementation of the intervention in real life thus necessarily entails difficulties and the improvement of professional practices in the MR process appears to be a key issue in ameliorating its effectiveness and efficiency. This work must be carried out with healthcare professionals working in primary care, i.e., GPs and CPs, hospital healthcare professionals as well as the patients and their potential caregivers in order to optimize the patient’s drug care pathway. Like some other studies, our results suggested that MRd has an impact on the way patients experience hospital discharge [44, 49]. Their involvement in identifying actions to improve the MR process is therefore essential.

Several points could thus be discussed to improve the MRd process. In addition to considering the most appropriate time to conduct the patient interview at discharge, as mentioned above, the intensity of the intervention could also be increased by strengthening the coordination of the drug care pathway between the hospital and primary care. For example, the hospital pharmacist could systematically call the CP and CP could pursue a close follow-up after hospital discharge [13, 47, 48, 50]. It is also essential to improve patient empowerment (in particular through the use of adapted and co-constructed tools and enhanced training for pharmacists in conducting pharmaceutical interviews, adopting an educational approach) and to involve the caregiver in the pharmaceutical interview at discharge if he or she is responsible for the patient's medication [51, 52]. Furthermore, the implementation of integrated care requires the use of a shared information system between the healthcare professionals involved in the patient's drug care pathway in both hospital and primary care, which is not currently the case in France [53]. Finally, an increase in the resources dedicated to MR would make it possible to guarantee continuity of care by hospital pharmacists or other professionals (nurses, pharmacy assistants) in order to increase the number of patients receiving MRd.

### Strengths and weaknesses of the study

To our knowledge, few studies have evaluated the specific impact of the MRd alone as defined by the NHA and mostly only on medication errors, but with very little or little evidence and none on the at-risk population aged 65 or more [13, 29–32]. One of the strengths of our study is that it was designed with a pragmatic approach whose objective, according to Ford et al., “is to inform a clinical or policy decision by providing evidence for adoption of the intervention into real-word clinical practice”, and which has been widely deployed over the last 20 years [33, 35, 54, 55]. First, our results highlight the difficulties of implementing an intervention in a real-life setting as pharmacists were only able to perform MRd for two-third of patients who received MRa. Patients were thus included in several medical and rehabilitation wards, in healthcare facilities of different size and status, the number of exclusion criteria was limited and the patients experienced different care pathways. Pharmacists and physicians involved in the intervention had the necessary knowledge and skills to conduct the MR process, but were not specifically trained to work together for the study, had varying levels of professional experience and varying amounts of time dedicated to the deployment of MR in their facilities.

Our intervention was planned according to NHA recommendations with a precise description of each MR step, but we observed flexibility in its actual implementation e.g., not all patients in the “MRa and MRd group” received a patient interview at discharge. Moreover, we chose a primary endpoint of clinical significance rather than an event without systematic impact on the patient, e.g., medication errors or medication discrepancies.

Pragmatic studies thus complement randomized clinical trials on this topic with consideration of the generalizability of the findings to routine clinical practice situations and contribute to our understanding of the real-life effectiveness of interventions [33, 35].

Our study also has several limitations. Unlike other studies that focus on MRa and MRd compared to no intervention, all the patients included in our study encompassed MRa that could reduce the potential difference between the two groups on the primary outcome. Furthermore, due to the observational and pragmatic design of this study, patients who received both MRa and MRd may not be comparable to patients who only received MRa, which could lead potential confounding bias. For example, we could hypothesize that the pharmacist who lack time may have prioritized the MRd he performed, for instance by targeting the most at-risk patients (risky drugs, polypharmacy, more complex medical situations, etc.). However, we found no statistical difference between the characteristics of patients in the two groups. Finally, only 441 of the 470 patients estimated in the sample size calculation were initially included in the study due to the limited funding of pharmacists dedicated to perform MR, the reporting schedule imposed by the funder, i.e. the French Solidarity and Health Ministry and recruitment took more time than expected due to the organizational constraints. These limitations are inherent in pragmatic studies design and we thus cannot rule out that a lack of power could be leading to a nonsignificant result.

## Conclusion

This study found that the MRd had no effect on healthcare utilization 30 days after discharge in patients over 65 years of age, but appeared to improve the patient's experience of continuity of care. Further multiprofessional studies associating primary care healthcare professionals, i.e., GPs and CPs, hospital healthcare professionals as well as the patients and their potential caregivers are needed to improve professional practices in the MR process. Optimizing the procedures for the pharmaceutical interview at discharge—the time at which it is carried out, the patient empowerment, the systematic involvement of the caregiver if he or she is responsible of the patient's medication, the tools used and the training of hospital pharmacists—would appear to interesting way to improve the effectiveness of MRd and the continuity of the drug care pathway between the hospital and primary care.

## Supplementary Information

**Additional file 1. **Supplemental tables

## Data Availability

The datasets used and/or analysed during the current study are available from the corresponding author on reasonable request due to the study sponsor's data privacy policy.

## Abbreviations

GP: General Practitioner
CP: Community Pharmacist
MRa: Medication Reconciliation on admission
MRd: Medication Reconciliation on discharge
NHA: National Health Agency
ADE: Adverse Drug Event
CI: Confidence Interval
OR: Odds Ratio
BPMH: Best Possible Medication History

## Footnotes

1 CONPARMED study on “Guaranteeing the continuity of the medical care pathway of the older patient: a territorial approach to clinical pharmacy” (“Garantir la CONtinuité du PARcours de soins MEDicamenteux du patient âgé: une approche territoriale de pharmacie clinique” in French).

2 Called *Soins de Suite et de Réadaptation* (SSR) in French.
