# Supplementary material for: Pharmacist-led medication reconciliation at patient discharge: a tool to reduce healthcare utilization? an observational study in patients 65 years or older
Source: BMC Geriatr. 2022 Jul 13;22:576. doi: 10.1186/s12877-022-03192-3 (PMC9281036; doi:10.1186/s12877-022-03192-3)
Supplement: Supplementary file 1 — Additional file 1. Supplemental tables [file 12877_2022_3192_MOESM1_ESM.docx]

| **Supplemental Table 1. Results on primary endpoint according to drugs included in BPMH** | | | | |  |
| --- | --- | --- | --- | --- | --- |
| **Drugs at admission** | ATC Class | Total  n=377 | **Death, unplanned rehospitalization or emergency visit for ADEs** | | p-value |
|  |  |  | Yes  n=29 | No  n=348 |  |
| Peripheral vasodilators, vasoprotectives and lipid modifying agents | C04 - C05 - C10 | 288 (76.4%) | 20 (69.0%) | 268 (77.0%) | 0.327 |
| Opioids and [other analgesics and antipyretics](https://www.whocc.no/atc_ddd_index/?code=N02B&showdescription=no) | N02A - N02B | 235 (62.3%) | 17 (58.6%) | 218 (62.6%) | 0.667 |
| Antiepileptics, psycholeptics and psychoanaleptics | N03 - N05 - N06 | 175 (46.4%) | 16 (55.2%) | 159 (45.7%) | 0.325 |
| Anti-thrombotic agents | B01 | 166 (44.0%) | 13 (44.8%) | 153 (44.0%) | 0.928 |
| Drugs for acid related disorders | A02 | 132 (35.0%) | 9 (31.0%) | 123 (35.3%) | 0.640 |
| Drugs for obstructive airway diseases | R03 | 68 (18.0%) | 10 (34.5%) | 58 (16.7%) | 0.016 |
| Cardiac therapy | C01 | 54 (14.3%) | 7 (24.1%) | 47 (13.5%) | 0.161 |
| Corticosteroid for systematic use | H02 | 50 (13.3%) | 7 (24.1%) | 43 (12.4%) | 0.086 |
| [Blood glucose lowering drugs](https://www.whocc.no/atc_ddd_index/?code=A10B&showdescription=no) (except insulins) | A10B | 47 (12.5%) | 2 (6.9%) | 45 (12.9%) | 0.557 |
| [Alpha-adrenoreceptor antagonists](https://www.whocc.no/atc_ddd_index/?code=G04CA&showdescription=no) | G04CA | 44 (11.7%) | 8 (27.6%) | 36 (10.3%) | 0.012 |
| Antibacterials for systemic use and antimycobacterials | J01 - J04 | 32 (8.5%) | 5 (17.2%) | 27 (7.8%) | 0.086 |
| Antineoplasic agents and endocrine therapy | L01 - L02 | 24 (6.4%) | 3 (10.3%) | 21 (6.0%) | 0.415 |
| Antigout preparation | M04 | 22 (5.8%) | 2 (6.9%) | 20 (5.7%) | 0.682 |
| Insulins and analogues | A10A | 17 (4.5%) | 2 (6.9%) | 15 (4.3%) | 0.382 |
| Antiparkinson drugs | N04 | 17 (4.5%) | 2 (6.9%) | 15 (4.3%) | 0.382 |
| Immunostimulants and immunosuppressants | L03 - L04 | 11 (2.9%) | 3 (10.3%) | 8 (2.3%) | 0.044 |
| BPMH: Best Possible Medication History, ADE: Adverse Drug Event, ATC: Anatomical Therapeutic Chemical Classification system | | | | | |

| **Supplemental Table 2. Description of Medication Reconciliation on admission** | | | | | | |  |
| --- | --- | --- | --- | --- | --- | --- | --- |
|  | **Total (n=377)** | | **Healthcare professional involved^a^** | | | |  |
| **1. Data collection and summary** | 376 (100.0%) | | Pharmacy student | | | 158 (42.0%) |  |
|  |  |  | Junior pharmacist | | | 55 (14.6%) |  |
|  |  |  | Senior pharmacist | | | 114 (30.3%) |  |
|  |  |  | Pharmacy technician | | | 49 (13.0%) |  |
| **2. Data summary** | 376 (100.0%) | | Pharmacy student | | | 141 (37.5%) |  |
|  |  |  | Junior pharmacist | | | 78 (20.7%) |  |
|  |  |  | Senior pharmacist | | | 118 (31.4%) |  |
|  |  |  | Pharmacy technician | | | 39 (10.4%) |  |
| **3. BPMH validation and transmission** |  | |  | | | |  |
| Verification of the MR process and confirmation of the consistency of the BPMH | 377 (100.0%) | | Junior pharmacist | | | 164 (44.0%) |  |
|  |  |  | Senior pharmacist | | | 209 (56.0%) |  |
| Pharmacist-physician collaborative meeting | 277 (74.3%) | | Junior pharmacist | | | 106 (39.0%) |  |
|  |  |  | Senior pharmacist | | | 163 (59.9%) |  |
|  |  |  | Junior physician | | | 77 (28.3%) |  |
|  |  |  | Senior physician | | | 99 (36.4%) |  |
| Identification of unintentional discrepancies and entry in patient medical record ^b^ | 197 (98.5%) | | Junior pharmacist | | | 93 (45.8%) |  |
|  |  |  | Senior pharmacist | | | 110 (54.2%) |  |
| Correction of the prescription by physician | 171 (87.7%) | | Junior physician | | | 83 (50.3%) |  |
|  |  |  | Senior physician | | | 82 (49.7%) |  |
| BPMH: Best Possible Medication History  Percentages were calculated excluding missing data.  ^a^ If several professionals were involved, the most qualified was considered.  ^b^ Among the 200 patients with at least one unintentional discrepancy | | | | | | |  |
| **Supplemental Table 3. Description of Medication Reconciliation on discharge** | | | | | | | |
|  | | **Total (n=221)** | | **Healthcare professional involved^a^** | | | |
| **1. Data collection and summary** | |  | |  |  | | |
| Patient chart review and current prescriptions | | 215 (100.0%) | | Junior pharmacist | 91 (42.5%) | | |
|  |  |  |  | Senior pharmacist | 123 (57.5%) | | |
| Data summary and comparison to BPMH on admission | | 213 (100.0%) | | Junior pharmacist | 90 (42.7%) | | |
|  |  |  |  | Senior pharmacist | 121 (57.4%) | | |
| **2. Data validation** | |  | |  |  | | |
| Pharmacist-physician collaborative meeting | | 185 (86.9%) | | Junior pharmacist | 70 (38.0%) | | |
|  |  |  |  | Senior pharmacist | 112 (60.9%) | | |
|  |  |  |  | Junior physician | 70 (38.0%) | | |
|  |  |  |  | Senior physician | 81 (44.0%) | | |
| Production of the discharge prescription by physician | | 204 (95.8%) | | Junior physician | 85 (42.9%) | | |
|  |  |  |  | Senior physician | 113 (57.1%) | | |
| Production of the MRd letter by pharmacist | | 215 (100.0%) | | Junior pharmacist | 87 (41.4%) | | |
|  |  |  |  | Senior pharmacist | 91 (43.3%) | | |
|  |  |  |  | Junior physician | 53 (25.2%) | | |
|  |  |  |  | Senior physician | 6 (2.8%) | | |
| **3. Data transmission** | |  | |  |  | | |
| Patient interview +/- patient’s informal caregiver | | 161 (74.9%) | | Junior pharmacist | 64 (42.4%) | | |
|  |  |  |  | Senior pharmacist | 87 (57.6%) | | |
| Production of the MRd letter | | 215 (100.0%) | |  |  | | |
| Sending MRd letter to GP | | 185 (87.3%) | |  |  | | |
| Sending MRd letter to community pharmacist | | 168 (79.2%) | |  |  | | |
| Production of the discharge prescription | | 204 (95.8%) | |  |  | | |
| Sending prescription to GP | | 8 (3.8%) | |  |  | | |
| Sending prescription to community pharmacist | | 48 (22.7%) | |  |  | | |
| GP: General Practitioner, MRd: Medication Reconciliation on Discharge, BPMH: Best Possible Medication History  Percentages were calculated excluding missing data  ^a^ If several professionals were involved, the most qualified was considered | | | | | | | |
